# DETERMINATION OF OPTIMAL VIBRATION DOSE TO TREAT PARKINSON’S DISEASE GAIT SYMPTOMS: A CLINICAL TRIAL

**DOI:** 10.1093/geroni/igad104.3739

**Published:** 2023-12-21

**Authors:** Ingrid Pretzer-Aboff, R K Elswick, Arnaud Gouelle, Noah Helm, GinaMari Blackwell, Leslie Cloud

**Affiliations:** Virginia Commonwealth University, Richmond, Virginia, United States; Virginia Commonwealth University, Richmond, Virginia, United States; Université Reims Champagne Ardenne, Reims, Champagne-Ardenne, France; Virginia Commonwealth University, Mechanicsville, Virginia, United States; Virginia Commonwealth University, Glen Allen, Virginia, United States; VCU School of Medicine, Henrico, Virginia, United States

## Abstract

Most people with Parkinson’s disease (PD) will experience gait problems. Previous studies demonstrated improved gait and balance after vibration stimulation was applied to the feet of PD patients. However, not all study participants showed improvement, perhaps due to sub-optimal vibration stimulus. Thus far, the optimal frequency and amplitude of vibration for mitigating gait dysfunction in PD have yet to be systematically explored. The PDVibe2TM (Resonate Forward LLC, DE) delivered vibration to the feet of 26 people with PD gait disturbances. We hypothesized that a global frequency, amplitude, and minimum duration of vibration therapy is required to improve gait. This was a phase Ib trial to identify optimal vibration parameters. Thirteen participants were recruited at Hoehn & Yahr (H&Y) stage II and 13 participants at stage III. Each group was randomly assigned to different frequency and amplitude settings prescribed by the central composite design methodology. Each participant received vibration for 18 minutes per walking session, for eight sessions spread over one week. Results showed an optimal dose response to treatment for frequency and amplitude of vibration based on the Functional Ambulation Performance score for stages II and III. In the H&Y stage II group, outcomes were stabilized after the 4th treatment. This stabilization was not seen in stage III participants. Global frequency and vibration amplitudes have been identified for treating PD gait disorders. Patients with more advanced Parkinson’s disease may require a longer duration of therapy.

